# GIMAP Proteins in T-Lymphocytes

**DOI:** 10.1155/2010/268589

**Published:** 2010-08-31

**Authors:** Sanna Filén, Riitta Lahesmaa

**Affiliations:** ^1^Turku Centre for Biotechnology, University of Turku and Åbo Akademi University, Tykistökatu 6 B, P.O. BOX 123, 20520 Turku, Finland; ^2^Department of Pharmacology, Drug Development and Therapeutics, Drug Discovery Graduate School, University of Turku, Itäinen Pitkäkatu 4 B, 20520 Turku, Finland

## Abstract

(GIMAPs) GTPase of the immunity associated protein family are a novel protein family of putative small GTPases. GIMAPs are mainly expressed in the cells of the immune system and have been associated with immunological functions, such as thymocyte development, apoptosis of peripheral lymphocytes and T helper cell differentiation. GIMAPs have also been linked to immunological diseases, such as T cell lymphopenia, leukemia and autoimmune diseases. In this review we examine the role of GIMAP proteins in T-lymphocyte biology.

## 1. GTPase of the Immunity-Associated Protein Family (GIMAP)

GTPase of the immunity-associated protein family (GIMAPs), also termed immune-associated nucleotide-binding proteins (IANs), are a relatively recently described, uncharacterized protein family of putative small GTPases conserved among vertebrates and higher plants [1–4]. The original publications describing the different human, mouse, and rat *GIMAP* family members used a variety of different names for the genes generating confusion. Thus, the *GIMAP* nomenclature followed also in this minireview was introduced by the HUGO Gene Nomenclature Committee and includes the human, mouse, and rat orthologs [5] (Table 1). 

Small GTP (guanosine triphosphate) binding proteins, also known as small GTPases, Ras-like GTPases, or Ras superfamily of GTP binding proteins, regulate key cellular functions in virtually all living organisms. They are involved in signal transduction events and regulation of gene expression in almost all cell types, including the cells of the immune system [26–28]. The Ras superfamily can be subclassified into Ras, Rho, Rab, and Arf families, and the closely related G_*α*_ family of the heterotrimeric G proteins, which sometimes are excluded from the RAS superfamily [29]. The Ras proteins induce signaling pathways that include a variety of second messengers, such as calcium and cAMP. The Ras superfamily proteins play key roles in a variety of cellular functions in the immune system, such as cell migration [30], T-cell anergy [31, 32], antigen presentation, [33] and radical formation [34]. 

The GIMAP family members have unique primary structures and, thus, they define a new family of G proteins distinct from the Ras superfamily and the heterotrimeric G proteins [1]. The expression of *GIMAP*s in vertebrates has been shown to be highest in the cells of the immune system, although a more ubiquitous expression has also been suggested. Several studies have associated GIMAPs with immunological functions, such as thymocyte development and apoptosis regulation in lymphocytes. These are discussed in what follows.

## 2. Genomic Organization of *GIMAP* Genes

**Figure 1:**
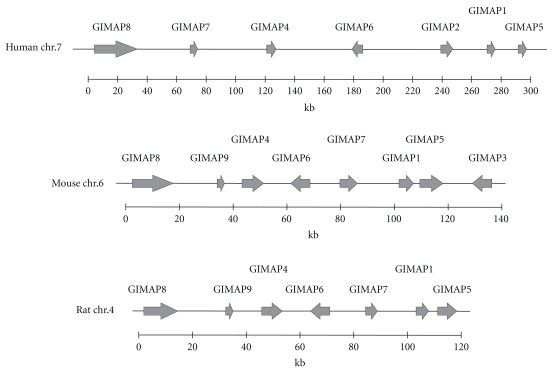
*GIMAP* gene clusters in human, mouse, and rat chromosomes. The *GIMAP* genes are clustered in human chromosome 7q36.1, mouse chromosome 6, and rat chromosome 4.

All vertebrate species examined so far have *GIMAP* genes in tight clusters in their genome [3, 4, 12]. The seven functional human *GIMAP* genes and one pseudogene are clustered on chromosome 7q36.1 [3] and there are eight functional mouse *Gimap*s clustered on chromosome 6 and seven functional genes in rat chromosome 4 [13, 25]. The ongoing sequencing project of the genome of *Danio rerio* (zebrafish) has revealed the existence of *Gimap* orthologs also in a lower vertebrate. The genomic organization of human, mouse and rat *GIMAP* genes is depicted in Figure 1. 

Homolog searches in available corn, soybean, and tobacco genomes by Liu et al. [4] came up with one to two homologs of *GIMAP/IAN* genes in each genome. However, searches within the well-characterized genomes of the unicellular organisms *Saccharomyces cerevisiae* (Baker's yeast) and *Schizosaccharomyces pombe *(fission yeast), or invertebrates, such as *Caenorhabditis elegans* (free-living roundworm) and *Drosophila melanogaster *(fruit fly) did not reveal any homologs of the *GIMAP* gene family [4]. Thus, *GIMAP* genes exist only in vertebrates and angiosperm (i.e., flowering) plants and the yet poorly characterized cellular functions of the GIMAP proteins are specific for vertebrates and higher plants.

GIMAP/IAN proteins emerged before plants and animals split into their own evolutionary paths [4]. Phylogenetic analyses of both protein and genomic sequences [3, 4] showed that human and mouse GIMAPs 1, 4, 5, 6, 7, and 8 form highly orthologous pairs, and, thus, suggest that a gene duplication event in a common ancestor of rodents and primates gave rise to these genes. The phylogenetic analyses by Liu et al. [4] place the *Arabidopsis* and rice IANs to a clade distinct from the mouse and human GIMAP proteins, thus indicating that the gene duplication events have taken place after the divergence of vertebrates and plants.

## 3. Features of GIMAP Proteins

Human GIMAP proteins are relatively small proteins with one GTPase domain. Their molecular sizes range from 34 kDa to 38 kDa. GIMAP8 makes an exception by having three GTPase domains, which is extremely unusual not only for GIMAPs, but for small GTPases in general, too. Thus, its molecular size is 74.9 kDa, making it by far the largest GIMAP protein. 

The GTPase domain with the five motifs G1-G5 characteristic for all small GTPases is included in the AIG1 domain, named after the prototype gene *AIG1* found in *Arabidopsis thaliana* (avrRpt2-induced gene) [35]. The AIG1 domain is found in all GIMAP and IAN proteins and besides the GTPase motifs, it contains a conserved box, which is characteristic for all AIG1 domain GTPases [3]. All human GIMAPs also contain putative coiled coil domains which suggest protein-protein interactions. Some GIMAPs, namely, GIMAP1, 2, 4, and 5, contain putative transmembrane domains in their COOH-terminal ends and GIMAP7, GIMAP6, GIMAP1, and GIMAP2 have basic amino acids in their NH_2_- or COOH-terminus with weak similarity to endoplasmic reticulum- (ER)-localization signals [3]. However, localization studies found GIMAP4 mainly in cytosol [12, 14] but also in ER and Golgi [3] and in membrane fraction of fractionated CD4^+^ T-lymphocytes [36]. GIMAP1 has been reported to localize in ER [6] and GIMAP7 in ER and Golgi [3]. 

Human GIMAP4 was shown to be able to bind guanosine nucleotides GTP and GDP but not GMP or other nucleotide triphosphates ATP, CTP or TTP [15]. GIMAP4 showed also intrinsic GTPase activity [15]. Besides the GTP, binding domain, GIMAP4 protein has other interesting motifs. It has a carboxy-terminal IQ domain, which is known to bind calmodulin, a second messenger molecule involved in, among other cellular processes, TCR signaling, and activation of NFAT in T-cells [14, 37]. The IQ motif usually consists of ~23 residues and contains the consensus sequence *hydrophob*QxxxRxxxxRxxxR/K, where *hydrophob* is a hydrophobic residue [14]. This motif was proven to be functional in murine GIMAP4 and, furthermore, seems to be absent from other GIMAP protein family members [14]. Also the putative phosphoylation sites of murine GIMAP4 were shown to be functional, since mouse GIMAP4 was phosphrorylated rapidly after 10 minutes of PMA/ionomycin stimulation in primary splenocytes [14]. The phosphorylation was mediated by PKC since it was inhibited by a PKC inhibitor, Rottlerin. Interestingly, this phosphorylation was diminished completely after 40–60 minutes of stimulation. Four of the six putative phosphorylation sites of mouse GIMAP4 are also found in human GIMAP4. Thus, GIMAP4 is a lymphocytic signaling molecule.

## 4. GIMAPs in T-lymphocyte Development and Function

### 4.1. GIMAPs in Thymocyte Development

**Figure 2:**
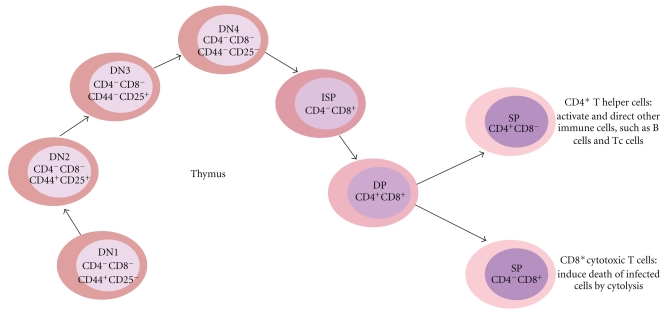
Thymocyte development. T-cells are developed in the thymus. The development can be defined by cell surface expression of CD4 and CD8. During the process, the thymosytes mature from CD4^−^CD8^−^ double negative (DN) cells into CD4^+^CD8^+^ double positive (DP) cells and finally into single positive (SP) CD4^+^ or CD8^+^ cells. The DN cells can be further categorized into DN1, DN2, DN3 and DN4 cells. ISP: immature single positive.

Cell surface expression of leukocyte markers CD4 and CD8 defines the stages of T-cell development in the thymus. During the development process, thymocytes mature from CD4^−^CD8^−^ double negative (DN) cells into CD4^+^CD8^+^ double positive (DP) cells and finally into single positive (SP) CD4^+^ or CD8^+^ cells which exit the thymus and enter the periphery (Figure 2). The DN cells are further categorized according to their CD44 and CD25 cell surface expression: (1) DN1 (CD44^+^CD25^−^), (2) DN2 (CD44^+^CD25^+^), (3) DN3 (CD44^−^CD25^+^), and (4) DN4 (CD44^−^CD25^−^). Furthermore, the DN4 cells pass through a CD4^−^CD8^+^ immature single positive (ISP) stage before developing into DP cells. The different maturation stages are phenotypically and functionally distinct. 

The expression of *GIMAP* genes is strictly regulated during T-cell development in the thymus. Dion et al. [13] studied the expression of rat *Gimaps* in different thymic subpopulations and observed a significant elevation in expression of all rat *Gimaps* but *Gimap 4* genes between the DN and DP stages. The expression patterns for *Gimap1*, *6*, *8,* and *9* during thymocyte development were very similar showing the highest expression in the DP stage. Mouse *Gimap 3, 4, 5, *and *7* expression was increased during the transition from CD4^+^CD8^+^ DP thymocytes into CD4^+^ or CD8^+^ SP thymocytes [12]. While shRNA mediated knockdown of mouse GIMAP4 hade no effect on thymocyte development in fetal thymic organ culture model shRNA mediated knockdown of mouse GIMAP3 and GIMAP5 disturbed thymocyte development [12]. Nitta et al. [12] showed that GIMAP3 was needed for optimal positive selection of SP CD4^+^ and CD8^+^ T-cells and Gimap5 was important for optimal development of DP thymocytes. It has also been shown that the proportion of CD4^−^CD8^+^ SP thymocytes was somewhat reduced in *Gimap5* ^−/−^ mice [38].

Expression of mouse GIMAP4 protein during thymocyte development has been studied in more detail by Poirier et al. [1] and Schnell et al. [14]. It was not detectable in DN1, DN2, DN3, or DP thymocytes whereas high levels of GIMAP4 were expressed in SP CD4^+^ cells and DN4 cells [1, 14]. The development of thymocytes in Rag^−/−^ mice is blocked at the DN3 stage but *in vivo* administration of anti-CD3 antibody allows a low number of thymocytes to proceed up to the DP-stage. This system allows for the enrichment of the low-frequency cell subsets from the different developmental stages, namely, DN3, DN4, and ISP, as well as DP cells. Using the Rag2^−/−^ model, Schnell et al. [14] demonstrated that mouse *Gimap4* transcript was absent in differentiation arrested Rag2^−/−^ DN3 thymocytes while it was present in high amounts in DN4, in lower amounts in ISP, and absent in DP cell subsets of anti-CD3 treated Rag2^−/−^ mice. Similar results were obtained by Poirier et al. [1] with a Rag^−/−^ fetal thymic organ culture (FTOC) model where thymocytes from Rag^−/−^ mice blocked at the DN3 stage were cultured *in vitro* with anti-CD3 antibodies. The antibody treatment resulted in the transient expression of GIMAP4 protein between the DN3 and DP developmental stages. The transitions both from DN3 to DN4 stages and from DP to SP stages require TCR-mediated signals, and, thus, the results described above indicate that GIMAP4 expression is directly or indirectly induced by TCR-mediated signals during thymocyte development. GIMAP4 was also shown to be upregulated by IL-7 signaling in mouse DN thymocytes [39]. However, GIMAP4^−/−^ mice showed no significant role for GIMAP4 in T-cell development in thymus [14]. Thus, GIMAP4 seems to be dispensable for T-cell development, although its expression is tightly regulated during the development process.

### 4.2. GIMAPs in Peripheral T-cell Functions

**Figure 3:**
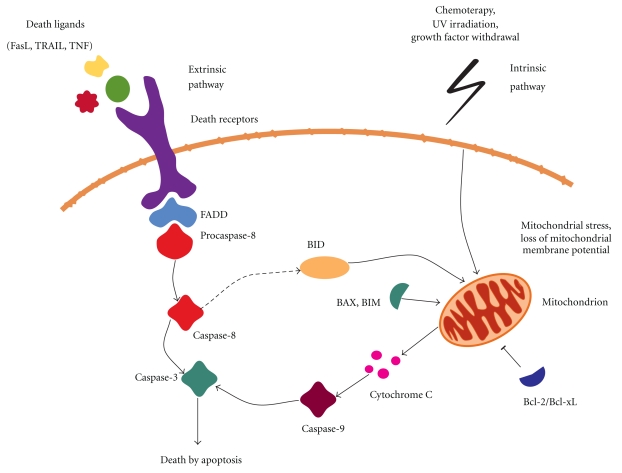
Apoptosis. Apoptosis can be initiated by the extrinsic or intrinsic pathways. The extrinsic pathway involves binding of ligands, such as tumor necrosis factor (TNF) or Fas ligand (FasL) to the death receptor. Subsequently, FAS-associating death domain (FADD) adapter protein is recruited to the receptor which leads to activation of caspase-8. The intrinsic pathway is initiated by cellular stress which induces loss of mitochondrial membrane potential, release of cytochrome C, and activation of caspase-9. The mitochondrial apoptotic pathway is controlled by the proapoptotic and antiapoptotic members of the Bcl-2 family. Cytochrome C release is inhibited by the prosurvival members Bcl-2/Bcl-xL and promoted by proapoptotic BAX/BIM. Both extrinsic and intrinsic pathways lead to activation of caspase-3 and, ultimately, cell death by apoptosis [40].

**Figure 4:**
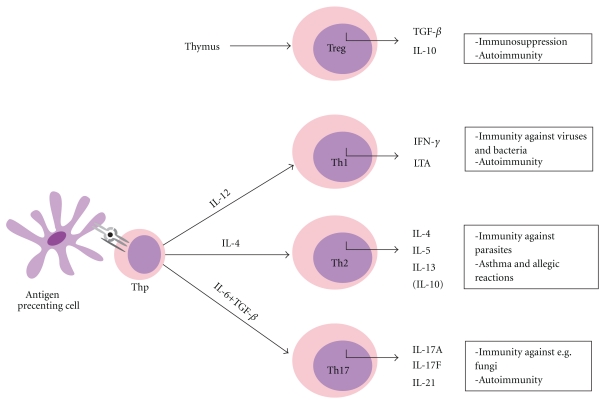
T-helper cell differentiation. Naïve Thp cells are able to differentiate into functional effector cell subsets according to the cytokine milieu. The differentiation process is initiated by antigen encounter. Naturally occurring regulatory T-cells are produced in the thymus. The different roles of the effector cells in the periphery are indicated in the figure.

Studies have indicated several roles for GIMAP proteins in peripheral T-lymphocyte functions. GIMAPs have been studied in most detail in apoptotic functions and in T-helper (Th) cell differentiation and these processes are depicted in Figures 3 and 4 and discussed below.

### 4.3. GIMAP1

Mouse *Gimap1* was first identified by Krücken et al. [7] as a gene expressed in the spleens of mice infected with *Plasmodium chabaudi *malaria. C57BL/10 mice are capable of self-healing the *P. chabaudi* malaria infection and this immunity is suppressed by testosterone. In the absence of testosterone the immunity progresses to a long-lasting protective immunity upon rechallenge. Once acquired, the immunity is testosterone independent and mediated by spleen cells [41]. The high expression of *Gimap1* in the spleen cells of *P. chabaudi* infected mice was strongly associated with the acquisition of the testosterone-resistant, immunity-mediating phenotype and was observed to be highest in macrophages with lower expression in B cells and T-cells [7]. Later it was shown that the expression of *Gimap1* in the spleens of the immune mice remains constitutively high for at least 13 weeks postinfection [8]. Surprisingly, however, a later study detected no upregulation of mouse GIMAP1 mRNA or protein in spleen cell lysates from mice infected with *P. chabaudi* [9] and this phenomenon could not be explained. Mouse *Gimap1* has also been reported to be upregulated by p53 during apoptosis in a mouse myeloid leukemia cell line LTR6 [10]. A conditional knockout of *Gimap1* in murine lymphocytes led to severe reduction of mature T and B cells but there was little effect on immature thymocytes [42]. Thus, GIMAP1 is critical for peripheral lymphocyte survival. 

Both human and mouse GIMAP1 orthologs are preferentially expressed in the spleen with some expression in the lymph nodes [6]. More detailed analysis of GIMAP1 protein expression in mouse splenic, thymic, and bone marrow cell populations revealed GIMAP1 expression in T (CD3+) and B (B220+) cells, DN, DP, and CD4+ and CD8+ SP thymocytes, as well as in NK/NKT (NK1.1+) cells [9]. Interestingly, GIMAP1 was expressed at significant levels in 5 out of 11 tested lymphoid cancer cell lines, namely C1498, TK-1, A20 and P815 [9]. 

It was shown that GIMAP1 was differentially expressed during human Th1/Th2 differentiation [36]. Its expression was induced by Th1 promoting cytokine IL-12 whereas its expression was downregulated by IL-4, a Th2-inducing cytokine. Promoter analysis of human *GIMAP1* gene revealed the existence of multiple silencer elements in the promoter [6]. Stamm et al. [6] studied a large 6.2 kb fragment containing also the first intron (from −3760 to +2419). Deletion of sequences from the first intron and from the 5′ flanking area resulted in inducible promoter activity indicating the removal of silencers. The smallest promoter clone of 0.8 kb (from −760 to +76) was highly active in Jurkat cells thus suggesting that it contained all the elements necessary for active transcription.

### 4.4. GIMAP3

*GIMAP3-ps* is a pseudogene in humans [3] and *Gimap3* is not annotated in the rat genome. Mouse *Gimap3* gene expression was detected at low levels in spleen, but no expression was detected in thymus, liver, or kidney [11]. However, high expression was detected in cell lines expressing BCR/ABL. BCR/ABL is a chimeric oncogenic protein generated from translocation between chromosomes 9 and 22 resulting in the so-called Philadelphia chromosome. BCR/ABL has constitutive tyrosine kinase activity and has been shown to activate several signal transduction pathways, such as RAS, RAF, MYC, and STAT [11]. Mouse GIMAP3 was also shown to bind GTP and localize in mitochondrial membranes [11].

### 4.5. GIMAP4

GIMAP4 protein expression is highly regulated at the post-transcriptional level [14, 15]. During T- and B-cell activation by anti-CD3/anti-CD28 and CD40-ligand/IL-4 stimulation, respectively, the mRNA level expression of *GIMAP4* was shown to be quite stable [15]. However, GIMAP4 protein level started to decrease after 4 days of T-cell activation and was undetectable at day 6. Similarly, yet more rapidly, B-cell activation resulted in the reduction of GIMAP4 protein already after two days of activation. The rapid activation induced phosphorylation/dephosphorylation/degradation of mouse GIMAP4 observed by Schnell et al. [14] also suggest a tight post-transcriptional regulation.

The expression of rat GIMAP4 protein was approximately 10-fold greater in wild type lymph node (LN) T-cells compared to LN T-cells from *lyp/lyp* (*Gimap5* ^−/−^) rat [13]. It is not known whether this is due to GIMAP4 and GIMAP5 interaction in some shared biochemical pathway or a result of the altered activation status of T-cells from *lyp/lyp* animals. The latter seems plausible in the light of the findings by Cambot et al. [15] and Schnell et al. [14] indicating that both human and mouse GIMAP4 protein expression decreased in response to activation. While the majority of the peripheral T-cells of wt animals are resting G_0_ cells, the T-cells in *lyp/lyp* rats are a mixture of semiactivated cells and recent thymic emigrants (RTE) [43, 44]. 

Although *Gimap4* knockout seemed to be redundant for T-cell development as well as selection and activation of T-cells *in vivo,* the knockout had a significant effect on T-cell apoptosis [14]. Apoptosis induces alterations in the plasma membrane composition and permeabilisation. A convenient method for measuring apoptosis is the staining of exposed phosphatidylserine (PS) on plasma membrane with fluorochrome labeled Annexin V and measuring the accumulation of propidium iodide (PI) in the nucleus after alterations in plasma membrane permeability. Apoptotic cells are, thus, PS positive and PI negative. When mature splenic T-cells from wild-type and *Gimap*4^−/−^ mice were induced to apoptosis, the number of apoptotic (PS^+^/PI^−^) cells was greater among the *Gimap*4^−/−^ T-cells than the wt T-cells and the number of dead cells were reduced accordingly, that is, the cells executed apoptosis with slower kinetics [14]. Furthermore, the accumulation of apoptotic cells could be inhibited by caspase inhibitors, and there were no changes in caspase-3 activation between apoptotic ko and wt T-cells. This indicated that GIMAP4 acts downstream of caspase-3 and plays a role rather in the execution than the induction phase of apoptosis. A similar, but less marked effect was found in T-cells of the inbred Brown Norway (BN) rat, which carries a natural hypomorphic variant of the *Gimap4 *gene [16]. Further support for the proapoptotic function of GIMAP4 came also from the findings of Nitta et al. [12], who showed that ectopic expression of GIMAP4 in immature mouse thymocytes led to increased apoptosis. Furthermore, GIMAP4 protein was shown to associate with the proapoptotic Bcl-2 family protein Bax but not other Bcl-2 family members [12, 45]. 

Although Schnell et al. [14] and Carter et al. [16] showed that GIMAP4 is largely a cytosolic protein Filén et al. [36] found it to be expressed in the membrane fraction of human CD4+ T-helper cells. GIMAP4 expression was furthermore shown to be tightly regulated during early human CD4+ T-helper cell differentiation towards Th1 and Th2 [36]. Proteomic analysis of IL-4 regulated membrane proteins revealed downregulation of GIMAP4 during early Th2 differentiation and it was further shown that IL-12 upregulated GIMAP4 expression during Th1 differentiation. The differential expression of *GIMAP4* was detected also on mRNA level. The expression of two distinct isoforms of *GIMAP4* in response to IL-12 and IL-4 was also studied and it was shown that they followed a similar expression pattern in response to the studied cytokines, although the short isoform was more highly abundant. RNAi studies further showed that GIMAP4 protein expression was negatively regulated by STAT6 in response to IL-4R signaling during Th2 differentiation [36].

### 4.6. GIMAP5

Human *GIMAP5* mRNA expression showed very wide tissue distribution among most human tissues outside the central nervous system [17, 18]. Its expression was upregulated during human peripheral T-cell activation [19]. It seemed to localize to the mitochondrial membrane [18] although localization to the centrosome/ER/plasma membrane [19] or to a distinct sedimentable subcellular fraction outside ER and mitochondria [46] has also been detected. The regulation of *GIMAP5* gene expression remains largely unknown but it has been shown that *GIMAP5* is a transcriptional target of Notch signaling in Jurkat cells [47]. 

An extensively studied genetic defect of lymphopenia (i.e., *lyp*, also called *Iddm2*) in diabetes-prone BioBreeding rat (BBDP) was shown to be caused by a frameshift mutation in the *Gimap5* gene [20–23]. This frameshift deletion of 1 bp introduces a premature STOP codon leading to a truncated protein product. This deletion was absent in the diabetes resistant BB (BBDR) rat [20, 21]. The BBDP spontaneously develop insulin-dependent diabetes mellitus (IDDM). It closely resembles human type 1 diabetes and is a consequence of Th1-mediated destruction of pancreatic islet *β* cells characterized by high levels of IFN-*γ* and IL-12 [48, 49]. The BBDP rat has severe, life-long T-cell lymphopenia [43, 50] caused by increased apoptosis and shortened life-span of recent thymic emigrants and peripheral T-cells [51–53]. However, the RTE can be rescued from apoptosis by activation through TCR [51]. Furthermore, peripheral *lyp/lyp* T-cells display normal Ca^2+^ signaling and proliferation in response to TCR crosslinking [52]. Interestingly, the *in vitro* life-span of *lyp/lyp* B cells is not affected by the mutation [52].

The lymphopenic phenotype of the BBDP rat argues for anti-apoptotic function for GIMAP5. The studies with the mutated GIMAP5 in BBDP rat by Pandarpurkar et al. [24], Keita et al. [46], and Pino et al. [54] and with human GIMAP5 by Sandal et al. [17] support this finding. Mitochondrial membrane potential was found to be lower in BBDP T-cells than in BBDR T-cells. The lack of full-length GIMAP5 in BBDP rat T-cells lead to ER stress signaling and C/EBP homologous protein-(CHOP)-mediated apoptosis [54]. Thus, GIMAP5 is a regulator of ER homeostasis in rat T-cells. The BBDP rat T-cells also display an impaired TCR-stimulated Ca^2+^ response which suggests that GIMAP5 is a regulator of calcium responses in T-lymphocytes [55]. Moreover, knockdown of *Gimap5* by RNAi in Jurkat T-cells resulted in increased apoptosis [24]. *Gimap5* ^−/−^ mice exhibit peripheral T-cell lymphopenia, especially of CD8^+^ T-cells [38]. The *Gimap5* ^−/−^CD8^+^ splenocytes furthermore showed increased apoptosis [38]. Overexpression of human *GIMAP5* in Jurkat T-cell line resulted in increased resistance to okadaic acid or *γ*-irradiation induced apoptosis. The anti-apoptotic function of GIMAP5 was shown to be upstream of caspase-3 [17]. However, more recent studies by Dalberg et al. [19] were contradictory to the earlier findings and showed a proapoptotic function for human GIMAP5. They showed that *GIMAP5* knockdown by RNAi did not affect the number of apoptotic cells but overexpression of *GIMAP5* in both Jurkat T-cells and naïve T-cells led to increased apoptosis [19]. Furthermore, they showed that while expression of the wt rat GIMAP5 led to increased apoptosis of the cells, the expression of the truncated GIMAP5-LYP in C58, a rat T-cell line, led to a very rapid death of the transfected cells [19]. This indicated a function for the truncated GIMAP5-LYP. Albeit the results obtained by different research groups seem contradictory, they may only indicate that the role of GIMAP5 in apoptosis is dependent on the activation status of the cells and the availability of growth factors. 

When the rat *Gimap5-Lyp* mutation was backcrossed into PVG-*RT1^u^* rat strain the *lyp/lyp* rats developed a spontaneous, progressive, inflammatory bowel disease with Th2 characteristics [56]. Indeed, the purified *lyp/lyp* CD4^+^CD45RC^lo^ cells produced increased amounts of IL-4 but similar amounts of IFN-*γ* compared to control wt cells. Also the expression of IL-4 and IL-13 was higher in the *lyp/lyp* cells compared to wt [56]. Accordingly, chemical mutation of mouse *Gimap5* led to severe intestinal inflammation and wasting disease [57]. 

A common polyadenylation polymorphism in the human *GIMAP5* gene was associated with the risk to autoimmune systemic lupus erythematosus (SLE) [58] and with the increased prevalence of IA-2 autoantibodies in patients with type I diabetes [59]. This polymorphism produces an inefficient polyadenylation signal to the 3′ part of the *GIMAP5* mRNA and leads to increased proportion of nonterminated mRNA [58].

## 5. Concluding Remarks

There are a growing number of reports describing a functional role for GIMAP family proteins in lymphocyte biology and we have discussed the role of GIMAPs in T-lymphocyte functions. More investigation is warranted to reveal the detailed molecular mechanism of GIMAP protein function. Determining, for example, the interaction partners, site of action, and signaling pathways will strengthen our knowledge of the function of this highly interesting novel family of GTPases.

## Figures and Tables

**Table 1:** Nomenclature of the human, mouse, and rat GIMAP genes and gene expression sites.

Gene	Synonyms, human	Synonyms, mouse	Synonyms, rat	Size kDa (human)	Expression	Reference
GIMAP1	GIMAP1, IMAP1, HIMAP1, IMAP38, hIan2	Gimap1, imap, IAP38, Imap38, mIan2	Gimap1, rIan2, Imap38, MGC156493	34.4	thymocytes, spleen, lymphocytes (T, B, NK), macrophages, cell lines (LTR6, C1498, TK-1, A20, P815)	[6–10]
GIMAP2	GIMAP2, IMAP2, HIMAP2, hIan12, MGC24275, DKFZp586D0824	no mouse ortholog	no rat ortholog	38.0	spleen, lymph nodes, PBL, thymus	[3]
GIMAP3	GIMAP3P (pseudogene)	Gimap3, mIan4, Gimap5, 2010110D23Rik	not annotated		spleen, cell lines expressing BCR/ABL	[11]
GIMAP4	GIMAP4, IAN1, IMAP4, hIan1, HIMAP4, MSTP062, FLJ11110	Gimap4, mIan1, IMAP4, mIAN1, AU019574, MGC11734, E430007K16Rik	Gimap4, rIan1	37.5	splenocytes, lymphocytes (T and B), thymocytes	[12–16]
GIMAP5	GIMAP5, IAN4, IAN5, IMAP3, hIan5, HIMAP3, IAN4L1, FLJ11296, Irod	Gimap5, mIan5, D630024P16, E230026N22Rik	Gimap5, IAN4, rIan5, Ian4l1	34.8	wide tissue distribution outside the central nervous system	[17–24]
GIMAP6	GIMAP6, hIan6, hIAN2, FLJ22690, DKFZp686A01175	Gimap6, mIan6, FLJ00102; MGC41522, mFLJ00102, 4833419H03Rik	Gimap6, rIan6, MGC108948	32.9	spleen, lymph nodes, lungs, placenta	[3]
GIMAP7	GIMAP7, IAN7, hIan7, MGC27027	Gimap7, mIan7, mIan3, MGC41480	Gimap7, rIan3, MGC108919	34.5	spleen, thymus, lymph nodes, PBL	[3]
GIMAP8	GIMAP8, IANT, hIAN6, MGC129545, DKFZp667I133, hIan11/10/9, hIan4/3/6	Gimap8, IAN9, Gm457, IMAP8, mIan9 mIan11/10/9	Gimap8, rIan11/10/9, IanT, MGC116406	74.9	thymus, spleen	[3, 12, 13, 25]
GIMAP9	orthologous to human GIMAP7	Gimap9, BB145400, A630002K24, mIan3 mIan7	Gimap9, MGC124918, rIan3		thymus, lymph nodes	[3, 13, 20]
aGIMAP10P	no human ortholog	Gimap10-ps (pseudogene), mIan8	no rat ortholog			[3]
